# Successful Surgical Management of Chronic Subdural Hematoma in a Patient With Severe Thrombocytopenia due to Aplastic Anemia: A Case Report

**DOI:** 10.1002/ccr3.73076

**Published:** 2026-07-01

**Authors:** Zhenghao Fu, Jiankan Lu, Xinqing Deng

**Affiliations:** ^1^ Department of Neurosurgery Guangdong Sanjiu Brain Hospital Guangzhou China; ^2^ Department of Neurosurgery Liwan Central Hospital of Guangzhou Guangzhou China

**Keywords:** aplastic anemia, chronic subdural hematoma, neurosurgery, platelet transfusion, thrombocytopenia

## Abstract

This case report describes the successful surgical management of a chronic subdural hematoma (CSDH) in a 45‐year‐old male with severe aplastic anemia (AA) and severe thrombocytopenia. The patient presented with worsening headaches, and imaging confirmed a progressive CSDH with midline shift. Despite a platelet count of 22 × 10^9^/L, preoperative transfusion increased it to 44 × 10^9^/L, enabling a minimally invasive burr‐hole drainage procedure. Postoperative platelet support and atorvastatin therapy facilitated recovery, with imaging confirming hematoma evacuation and midline restoration. The case highlights the feasibility of neurosurgical intervention in severely thrombocytopenic patients through meticulous perioperative planning, including platelet management and minimally invasive techniques. It also underscores the potential role of atorvastatin in CSDH management, though further research is needed in thrombocytopenic populations. This report demonstrates that with careful perioperative planning, appropriate platelet support, and minimally invasive surgical technique, successful management of CSDH is achievable even in patients with severe thrombocytopenia secondary to AA.

## Key Clinical Message

Successful surgical management of chronic subdural hematoma in severe thrombocytopenia requires comprehensive preoperative preparation including platelet transfusion, a multidisciplinary approach, careful surgical technique, and intensive postoperative monitoring. This case demonstrates that neurosurgical intervention can be safely performed in aplastic anemia patients with appropriate hematological support and risk stratification.

## 1 Introduction

Aplastic anemia (AA) is a life‐threatening bone marrow failure disorder characterized by pancytopenia and hypocellular bone marrow [1]. The pathogenesis involves immune‐mediated destruction of hematopoietic stem cells, leading to bone marrow failure and subsequent pancytopenia. Severe thrombocytopenia (platelet count < 20 × 109/L) is a common manifestation that significantly increases bleeding risks [1, 2].

Chronic subdural hematoma (CSDH) is a common neurological disorder, with a mortality rate as high as 42% and a recurrence rate up to 76% [3]. The reported incidence of intracranial hemorrhage in AA patients ranges from 4% to 19.2%, with CSDH accounting for approximately 8.57% [4, 5, 6]. Surgical treatments are still the main solutions for progressive hematomas, including conservative treatment, twist drill or burr‐hole surgery, and craniectomy [7]. The management of CSDH in thrombocytopenic patients presents unique challenges, particularly regarding the timing of surgical intervention.

We present a rare case of successful surgical management of CSDH in a patient with severe AA, demonstrating that appropriate perioperative platelet management can enable safe neurosurgical intervention even in severely thrombocytopenic patients. This case provides valuable insights into the management strategies for this challenging clinical scenario.

## 2 Case Presentation

A 45‐year‐old male presented to our neurosurgical department with worsening headaches. He had a 4‐year history of AA with severe thrombocytopenia, managed with multiple medications including leucogen Tablets (20 mg orally three times daily), compound zaofan Pills (9 pills orally three times daily), cyclosporine (100 mg orally twice a day), and testosterone undecanoate soft capsules (80 mg orally once a day) for platelet production, along with carbazochrome tablets (5 mg orally three times daily) for bleeding prophylaxis.

The patient initially developed headaches 1 month before admission without a clear history of trauma, at which time computed tomography (CT) revealed a left fronto‐temporo‐parieto‐occipital chronic subdural hematoma (CSDH) without midline shift (Figure 1A). Three days prior to admission, his headache significantly worsened. Follow‐up CT demonstrated an enlarged left fronto‐temporo‐parieto‐occipital CSDH with a 1 cm midline shift and signs of herniation (Figure 1B).

**FIGURE 1:**
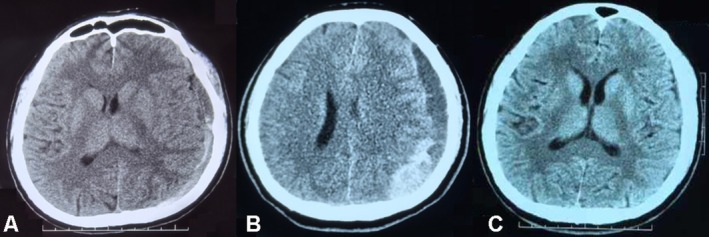
CT images of left frontotemporal‐parietal CSDH. (A) 1 month before admission. (B) 3 days prior to admission. (C) two‐month postoperative follow‐up.

Upon admission, laboratory studies revealed severe thrombocytopenia with a platelet count of 22 × 10^9^/L and significant anemia with hemoglobin at 60 g/L. Comprehensive coagulation studies were performed preoperatively and postoperatively. No coagulation abnormalities were observed through routine coagulation profile. Preoperative coagulation parameters: PT: 11.1 s (normal: 9.8–12.1 s), APTT: 27.7 s (normal: 25–31.3 s), Fibrinogen: 3.7 g/L (normal: 2–4 g/L), INR: 0.96 (normal: 0.85–1.05 g/L). Postoperative (24 h post‐surgery): PT: 11.0 s (normal: 9.8–12.1 s), APTT: 28.5 s (normal: 25–31.3 s), Fibrinogen: 4.8 g/L (normal: 2–4 g/L), INR: 0.95 (normal: 0.85–1.05 g/L).

Given the patient's critical neurological status and imaging findings confirming progressive CSDH with significant mass effect, immediate intervention was deemed necessary despite the severe thrombocytopenia. The patient received preoperative transfusion support with one therapeutic unit (approximately 3.0 × 10^11^ platelets from single‐donor apheresis platelets) of type O Rh‐positive platelets and 400 mL of red blood cells, which successfully increased his platelet count to 44 × 10^9^/L and hemoglobin to 75 g/L (Table 1). All blood components administered were leukoreduced. The RBCs were filtered using inline leukoreduction filters (Pall RC2D), and platelets were obtained from apheresis donations which are inherently leukoreduced.

**TABLE 1:** Changes in platelet count after platelet transfusion during the perioperative period.

Test	Results			Normal range
	Initial values at admission	Platelet transfusion before surgery	Platelet transfusion after surgery	
RBC	1.75	2.24	2.13	4.3–5.8 × 10^12^/L
Hemoglobin	60	75	72	120–160 g/L
Platelets	26	44	70	100–300 × 10^9^/L

Following optimization of his hematological parameters, we proceeded with a left temporo‐parietal single burr hole drainage procedure for hematoma evacuation. The scalp incision was 4 cm in length, and a single burr hole of approximately 1.5 × 1.5 cm was created. The dura mater and hematoma membrane were incised, followed by irrigation of the hematoma cavity with normal saline. A drainage tube was placed in the subdural space. We used prophylactic antibiotics with the following regimen: cefuroxime 1.5 g intravenously 30 min before surgery, then every 8 h postoperatively for 24 h.

Postoperatively, one therapeutic unit of platelets was administered, achieving a platelet count of 70 × 10^9^/L (Table 1). The patient's previous medications were continued, and Atorvastatin Calcium Tablets (20 mg per day, oral.) were added to the regimen. Postoperative CT on Day 3 demonstrated successful evacuation of the hematoma with restoration of midline anatomy and resolution of herniation signs, allowing for removal of the drainage tube.

The patient's recovery was complicated by mild epistaxis on postoperative Day 5, necessitating transfer to the hematology department for specialized management. Continue taking atorvastatin calcium tablets (20 mg per day) based on the treatment of AA. No adverse effects were observed during atorvastatin use. Liver function tests were monitored and remained within normal limits throughout the treatment period. At the 2‐month postoperative follow‐up, repeat CT imaging revealed complete resolution of the CSDH (Figure 1C). This case demonstrates that successful surgical management of CSDH is feasible in patients with severe AA through careful perioperative planning and appropriate blood product support, even in the setting of severe thrombocytopenia.

## 3 Discussion

The successful management of CSDH in patients with severe AA presents unique challenges that require careful consideration of medical therapy, surgical timing, and perioperative management. The successful management of chronic subdural hematoma in severe thrombocytopenia requires a multidisciplinary approach involving hematology and neurosurgery teams. Preoperative preparation included platelet transfusion to bring the platelet count close to 50,000/μL, comprehensive coagulation assessment, and optimization of the patient's hematological status. The surgical strategy emphasized minimal invasiveness with single burr‐hole drainage, meticulous hemostasis, and gentle technique to minimize bleeding risk. Postoperative management included intensive monitoring, prophylactic antibiotics due to immunosuppression, continued hematological support, and close neurological surveillance, ultimately achieving excellent functional recovery without complications. The favorable outcome in our case suggests that minimally invasive techniques, when combined with appropriate platelet support, can safely achieve therapeutic goals even in AA patients with severe thrombocytopenia.

Our surgical strategy emphasized the critical importance of timing and platelet management. The decision for surgical intervention was driven by progressive neurological deterioration and development of midline shift, balanced against the risks of perioperative bleeding. The burr hole surgery that involved hematoma evacuation and drainage is the most widely used approach [8]. The selection of single burr‐hole drainage over craniotomy represented a careful balance between effective hematoma evacuation and minimizing surgical trauma. This approach provided adequate decompression while reducing the risk of perioperative bleeding complications.

Current guidelines suggest that platelet counts should be maintained above 50 × 10^9^/L for most major surgeries, while for intracranial surgery platelet counts should be maintained above 70 to 100 × 10^9^/L [9], as a platelet count of ≥ 100 × 10^9^/L is required for platelet plug formation and therefore normal bleeding time [10]. Patients with platelet dysfunction have 2.74 times higher risk of CSDH recurrence compared to patients with normal platelet function [3]. However, recent studies have demonstrated successful outcomes with lower thresholds (28 × 10^9^/L) when combined with appropriate platelet support [11]. This is particularly relevant for emergency situations where immediate intervention is required despite suboptimal platelet counts. Our platelet transfusion strategy targeted a pre‐operative count exceeding 40 × 10^9^/L, achieved through careful timing of transfusions. This threshold, while lower than traditionally recommended for neurosurgical procedures [9], proved adequate when combined with minimally invasive surgical techniques during emergency situations. In addition, after relieving cerebral herniation with drainage surgery, continuous infusion of platelets was also crucial for reducing perioperative bleeding risk, especially for patients who responded well to platelet transfusion [12].

Emerging evidence suggests that atorvastatin may play a beneficial role in reducing CSDH recurrence [13]. The proposed mechanisms include anti‐inflammatory and anti‐angiogenic properties, enhancing endothelial progenitor cell function, thereby maintaining vascular membrane integrity to reduce vascular leakage and hematoma progression [14, 15, 16, 17]. The timing of atorvastatin administration appears crucial, with evidence suggesting maximal benefit when initiated at initial CSDH diagnosis. A RCT study has demonstrated significant reduction in hematoma volume and recurrence rates with atorvastatin treatment, particularly when administered early in the disease course [17]. Current literature advocates for a daily dosing regimen of 20 mg, although the optimal treatment duration remains controversial, ranging from 3 days to 6 months [13]. In our case, the patient maintained continuous medication therapy for AA, while simultaneously adhering to a 2‐month post‐operative course of atorvastatin calcium tablets. Follow‐up CT scan showed that the hematoma had completely disappeared, suggesting that atorvastatin may be used for preventing recurrence of CSDH in patients with AA. Post‐operative management focused on maintaining adequate platelet counts while monitoring for potential complications. The minor epistaxis observed post‐operatively was successfully managed with conservative measures, suggesting an acceptable risk profile for our approach. However, the safety and efficacy of atorvastatin in patients with severe thrombocytopenia secondary to AA require further investigation, as existing studies have largely excluded this population.

This case extends current understanding of CSDH management in severely thrombocytopenic patients, demonstrating that successful surgical intervention is possible with appropriate patient selection and perioperative management. The experience emphasizes the importance of early recognition of neurological symptoms in AA patients and suggests that prompt surgical intervention, when indicated by clinical deterioration, can be safely accomplished with adequate platelet support. This patient with AA successfully underwent neurosurgical intervention with appropriate hematological support; haemopoietic stem cell transplant should be considered as a standard first‐line treatment for severe/very severe AA patients [18], unfortunately, the patient currently has no plans for hematopoietic stem cell transplantation due to the high treatment costs.

Several limitations should be acknowledged in this case report. First, point‐of‐care coagulation tests such as ROTEM or TEG were not available, limiting our ability to assess global hemostatic function in real‐time; such tests should be performed to provide valuable additional hemostatic information in future similar cases.

Future research directions should address optimization of atorvastatin therapy in thrombocytopenic patients, refinement of platelet transfusion protocols for neurosurgical procedures, and long‐term outcomes of minimally invasive approaches in this challenging patient population. Additionally, whether this treatment strategy is applicable to AA patients who do not respond to platelet transfusion requires further exploration in the future.

## Author Contributions

**Zhenghao Fu:** conceptualization, data curation, formal analysis, methodology, project administration, software, visualization, writing – original draft, writing – review and editing. **Jiankan Lu:** data curation, formal analysis, investigation, methodology, validation. **Xinqing Deng:** conceptualization, data curation, formal analysis, investigation, methodology, project administration, resources, software, supervision, writing – review and editing.

## Funding

The authors have nothing to report.

## Consent

Written informed consent was obtained from the patient for publication of this case report and any accompanying images. A copy of the written consent is available for review by the Editor of this journal.

## Data Availability

The data that support the findings of this study are available from the corresponding author upon reasonable request.
